# Synchronous bilateral multifocal basal cell adenomas of the parotid gland—a case report

**DOI:** 10.1186/s12903-022-02339-3

**Published:** 2022-07-29

**Authors:** Jakub Piątkowski, Ewa Garsta, Grzegorz Śmigielski, Karolina Markiet, Bartosz Wasąg, Aleksandra Ciarka, Bogusław Mikaszewski

**Affiliations:** 1 Department of Otolaryngology, Faculty of Medicine, Medical University of Gdańsk, 17 Smoluchowskiego Street, 80-214 Gdańsk, Poland; 2 2nd Department of Radiology, Faculty of Health Sciences, Medical University of Gdańsk, 17 Smoluchowskiego Street, 80-214 Gdańsk, Poland; 3 Department of Biology and Medical Genetics, Faculty of Medicine, Medical University of Gdańsk, 1 Dębinki Street, 80-211 Gdańsk, Poland; 4 Department of Otolaryngology, University Clinical Centre Gdańsk, 17 Smoluchowskiego Street, 80-214 Gdańsk, Poland; 5 Department of Patomorphology, University Clinical Centre Gdańsk, 17 Smoluchowskiego Street, 80-214 Gdańsk, Poland

**Keywords:** Basal cell adenoma, Bilateral, Synchronous, Parotid gland, Case report

## Abstract

**Background:**

Bilateral parotid gland tumors account for up to 3% of cases. In this group, the vast majority are Warthin’s tumors. However, bilateral presentations of other parotid gland tumor entities is also possible, an example of which is a basal cell adenoma (BCA). Bilateral BCA is extremely rare, which could cause misdiagnosing it as a Warthin tumor.

**Case presentation:**

The current study reports the unique case of a 48-year-old woman who presented with a 6-month history of slowly growing masses located bilaterally in the parotid region, surgically treated with 5-year follow-up (no recurrence, normal facial nerve function). Magnetic resonance imaging (MRI) revealed three lesions: two in the superficial and deep lobes of the right parotid gland, and one in the superficial lobe of the left parotid gland. A total parotidectomy with facial nerve preservation was performed on the right side, and superficial parotidectomy on the left side 6 months later. Histopathological examination confirmed that all three tumors were BCAs. Molecular analysis didn’t show any strong, potential of unknown clinical significance in the studied sample.

**Conclusions:**

Multifocal bilateral lesions of the parotid gland are usually Warthin tumors. Detailed preoperative diagnostics including MRI and histopathological examination is essential to avoid misdiagnosing BCA and Warthin tumors. To our best knowledge, no case of synchronous bilateral multifocal basal cell adenomas of the parotid gland has been reported in English literature so far.

## Background

Salivary gland tumors (SGTs) constitute from 2 to 6.5% of head and neck tumors with many histopathological variants [1]. The WHO in 2017 described 42 histological types of SGTs, of which 11 are classified as benign tumors [2]. Basal cell adenoma (BCA) of the parotid gland was described first by Kleinsasser and Klein in 1967 [3]. These tumors are benign, accounting for 1–2% of all SGTs, with a female predominance of 2:1, occurring commonly in the fifth decade of life [4]. Basal cell adenoma is most often recognized within the parotid gland (70%) [5, 6]. Clinically BCAs are slowly expanding, asymptomatic, predominantly accidentally diagnosed. Bilateral occurrence of parotid gland tumors is seen in 1–3%, which of the vast majority are Warthin’s tumors [7]. Only a few cases of bilateral BCA’s have been reported in the literature so far. The current study reports a rare case of synchronous bilateral multifocal basal cell adenoma of the parotid gland in a 48-years-old woman.

## Case presentation

**Fig. 1:**
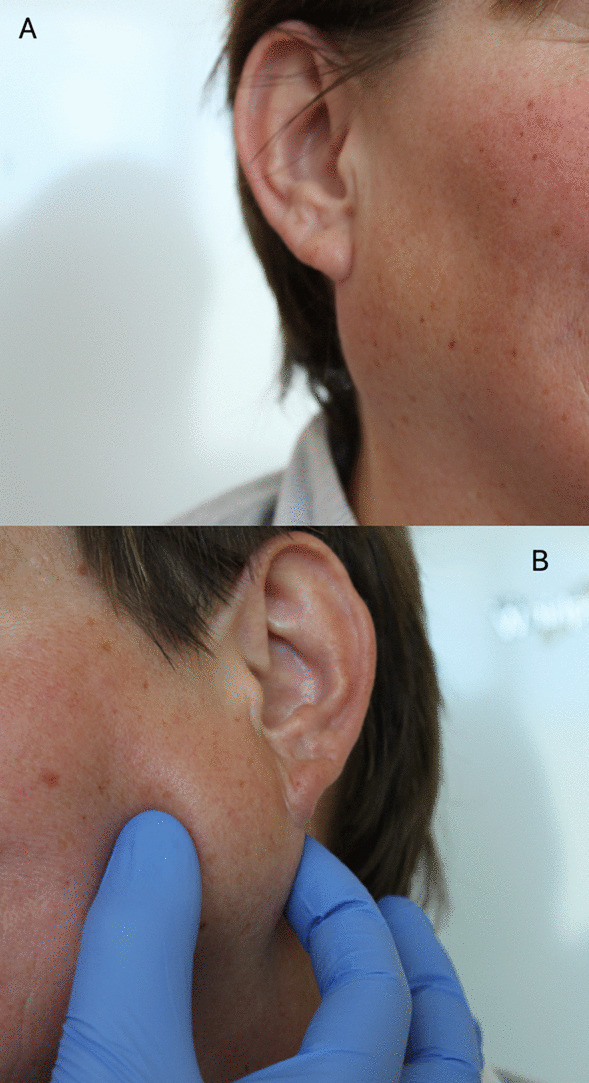
Patient with bilateral parotid gland tumors (**A**—right parotid gland tumor, **B**—left parotid gland tumor)

A 48-years-old woman was referred to The Department of Otolaryngology of the Medical University of Gdańsk (Poland) with palpable masses in the parotid region that have been slowly growing bilaterally for the last six months. There were no other symptoms and no relevant medical, family or psychosocial history. Facial nerves function was normal. Physical examination revealed the presence of firm, movable, bilateral parotid gland tumors, measuring 2 × 1.5 cm on the right side and 3 × 3.5 cm on the left side, with no signs of discharge from the salivary ducts. Moreover, no lymphadenopathy was observed (Fig. 1). A fine-needle aspiration biopsy reports were twice described as non-diagnostic.

**Fig. 2:**
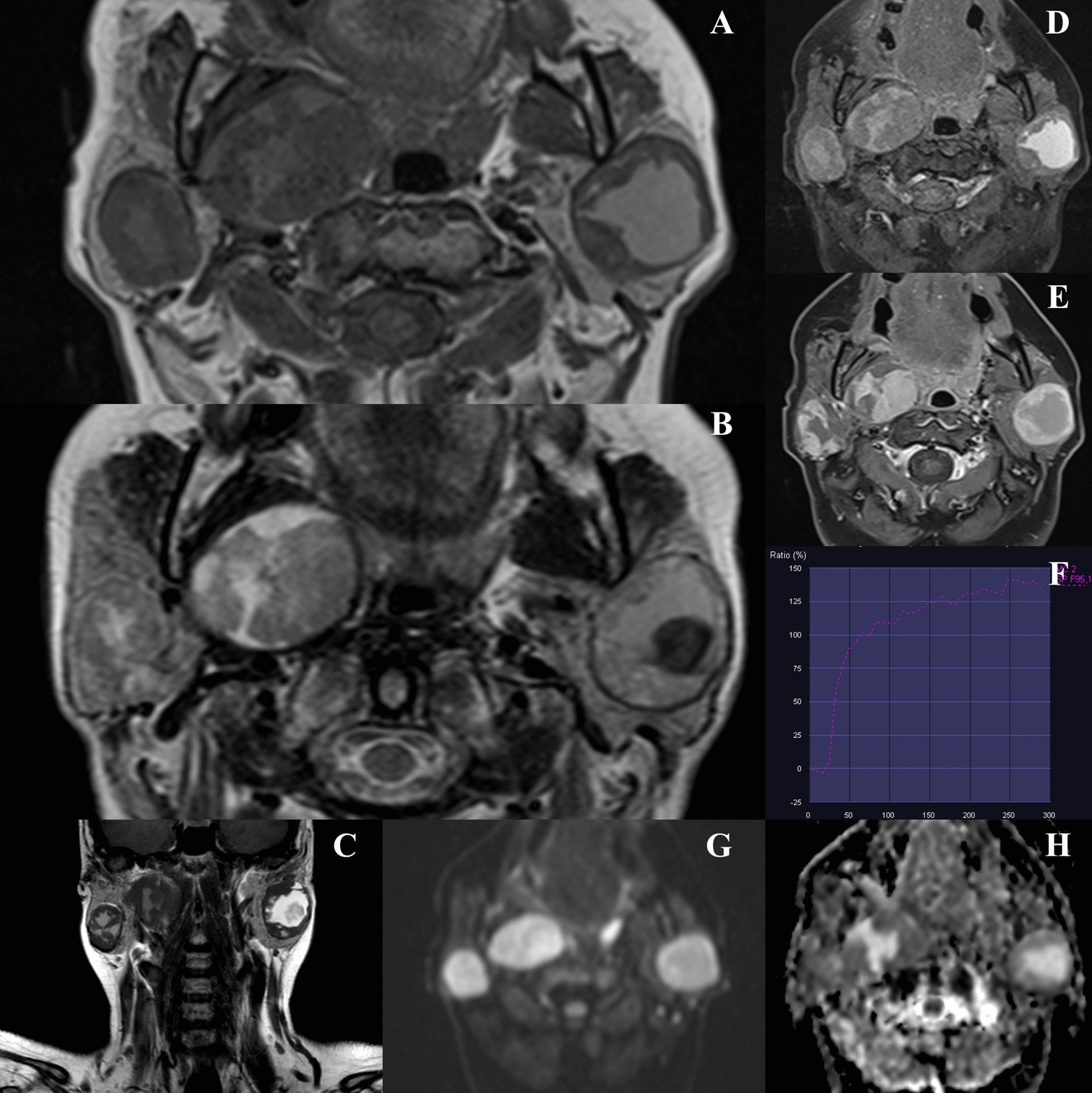
MRI of bilateral multifocal parotid basal-cell adenomas. The examination shows heterogeneous lesions, with central cystic areas, partially with higher protein content and signs of bleeding, and peripheral solid components (**A**. T1-weighted axial image, **B**. T2-weighted axial image, **C**. T2-weighted coronal image and **D**. T2-weighted fat-saturated axial image). Solid components of the tumours present vivid enhancement post-gadolinium-based contrast agent (**E**. contrast-enhanced T1-weighted fat-saturated axial image) and the time-intensity curves obtained on the basis of dynamic contrast-enhanced sequence show a continuous uptake of the contrast agent suggestive of adenomas (**F**). Tumors did not show signs of diffusion restriction with ADC values of approximately 1.48 × 10^−3^ mm.^2^/s (**G**. diffusion-weighted axial image, b 1000, H. ADC map)

**Fig. 3:**
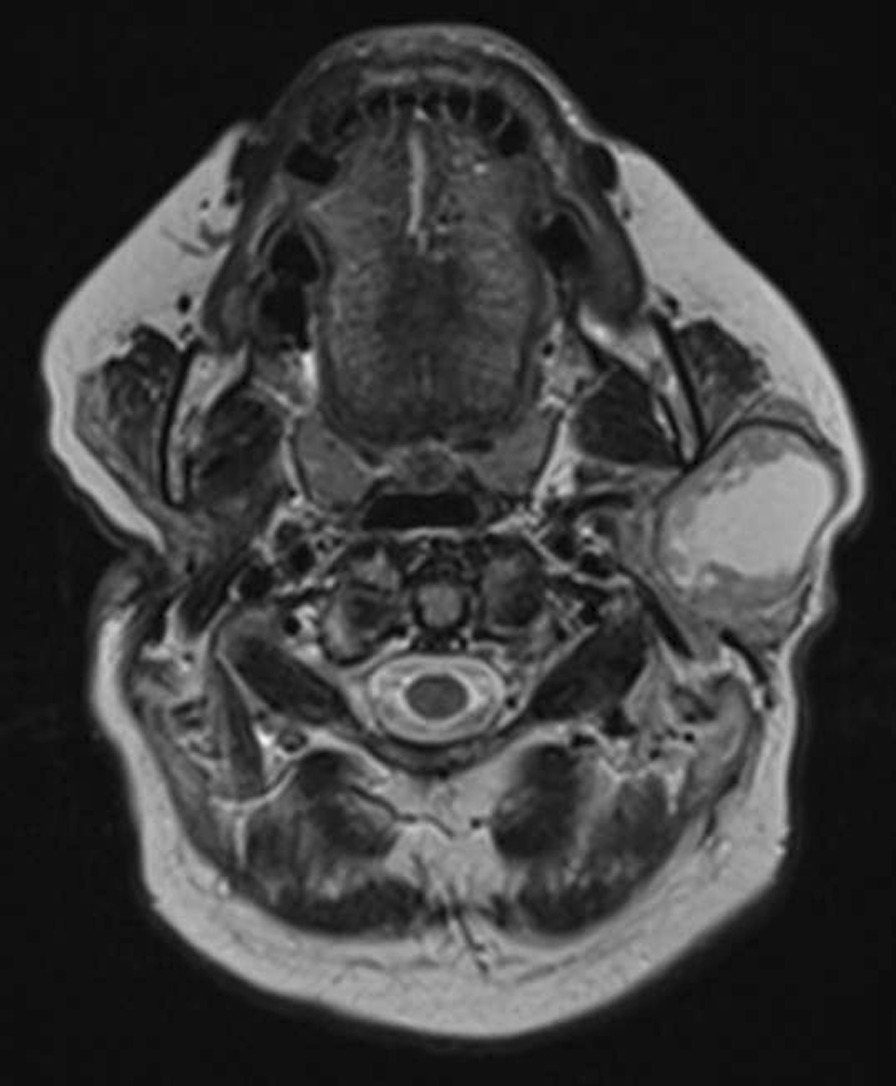
Axial T2-weighted MRI image post right total parotidectomy presenting a standard view of the post-operative site with one remaining lesion in the superficial lobe of the left parotid gland

Magnetic resonance imaging (MRI) with an application of standard T1 and T2-weighted sequences as well as diffusion-weighted imaging (DWI) and dynamic contrast-enhanced T1-weighted fat-saturated sequences was performed. The examination confirmed the presence of tumors within the superficial lobe of the left parotid (3.6 × 3.2 × 3.7 cm) and the inferior part of the superficial lobe of the contralateral parotid (2.9 × 2.6 × 3 cm). It revealed the second lesion in the deep lobe of the right parotid gland (4.8 × 3.6 × 4 cm) extending into the parapharyngeal space (Fig. 2). All three lesions had smooth, well-defined margins. They presented as heterogeneous structures, with central cystic areas, partially with higher protein content and signs of bleeding, and enhancing, more peripherally placed solid components. The time-intensity curves obtained based on dynamic contrast-enhanced sequence showed a continuous uptake of the contrast agent suggestive of adenomas. Tumours did not show signs of diffusion restriction with apparent diffusion coefficient (ADC) values of approximately 1.48 × 10^−3^ mm^2^/s (Fig. 3).

A total parotidectomy was performed on the right side with facial nerve preservation and resection of the two tumors in the superficial and deep lobe. The left side was operated on in the second stage, in 6 months—patient underwent superficial parotidectomy with tumor resection and facial nerve preservation.

**Fig. 4:**
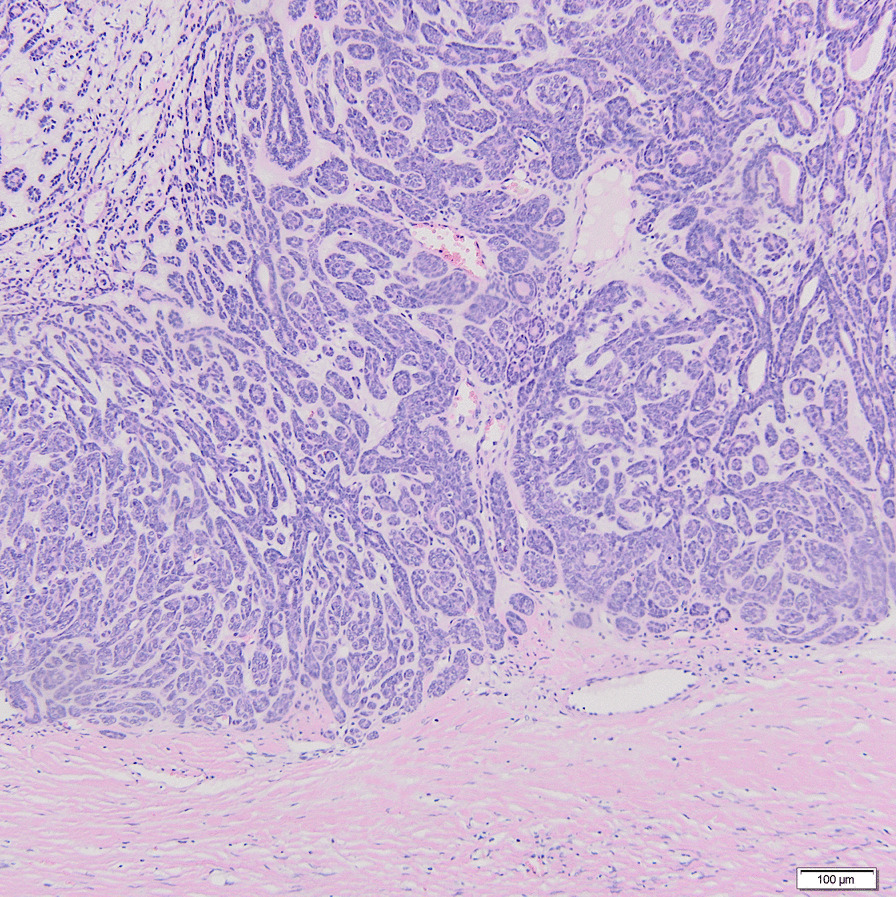
The tumor is surrounded by a fibrous capsule without evidence of invasion, as is characteristic of adenoma. This tumor shows architecture of basal cell adenoma (membranaceous growth pattern)—typical jigsaw puzzle–like islands of basaloid cells, interspersed by occasional vacuolar or small cystic spaces, the cell islands are sharply demarcated from the stroma (H&E staining, × 10)

Histopathological examination proved that all tumors were basal cell adenomas (membranaceous growth pattern). The tumors were surrounded by a fibrous capsule without evidence of invasion, as is characteristic for adenoma, with the following immunophenotype pattern—p63 protein (+), S-100 protein (±), epithelial membrane antigen (EMA) (+), CD117 protein (±), smooth muscle actin (SMA) (+). The excision of the tumors was complete (Fig. 4).

Finally, molecular analysis was performed. For this purpose, both DNA and RNA were isolated from formalin-fixed paraffin-embedded tissue, and Illumina TruSight Oncology 500 targeted hybrid-capture assay was used for comprehensive genome profiling. Libraries were sequenced using a NextSeq550 instrument (Illumina), and data were analyzed using the Clinical Genomics Workspace (PierianDx). No variants of strong, potential of unknown clinical significance were detected in the studied sample.

After 5-year of follow up, there were no clinical and ultrasonographical signs of recurrence. Facial nerves function was normal after surgery, as well as two years after the surgical procedure.

PubMed, Medline, Web of Science were searched for English-literature case reports and case series of synchronous basal cell adenoma of the parotid gland in the last 30 years. The following keywords were used: (Synchronous basal cell adenoma or Synchronous basal cell adenomas) and (Parotid gland or Parotid glands). We found eight reports presenting multifocal basal cell adenomas of the parotid gland (five synchronous, three metachronous) [8–10, 14–18]. Only five cases were included for analysis: four cases of synchronous bilateral basal cell adenomas and one case of synchronous unilateral basal cell adenomas. The literature results are presented in Table 1.

**Table 1:** Results of literature review on synchronous BCA of the parotid gland

Authors	Year	Synchronous multifocal unilateral	Synchronous bilateral monofocal	Synchronous bilateral multifocal
Kuang et al. [14]	2014	+	−	−
Reddy et al. [18]	2008	−	+	−
Suzuki et al. [17]	2000	−	+	−
Katsuno et al. [16]	2000	−	+	−
Zarbo et al. [15]	1985	−	+	−

## Discussion and Conclusions

Synchronous parotid gland tumors occur very rarely, with an incidence rate of less than 1% [11]. The most common multifocal growing lesion within the parotid is Warthin’s tumor [12, 13]. Basal cell adenomas are uncommon tumors of the salivary glands. BCA’s are oval-shaped, firm, slowly growing tumors in physical examination, usually less than 3 cm in size at diagnosis.

Synchronous BCA’s have been reported in only five cases: four had synchronous bilateral growth, and one had synchronous unilateral growth [14–18]. This study presents a unique case where both synchronous bilateral and multifocal growth of BCA’s occurred simultaneously. To the best knowledge of the authors, no similar case was reported previously.

The differential diagnosis of BCA of the parotid gland should include others pleomorphic adenoma (PA) and Warthin’s tumor. BCA in most cases, occurs unilaterally. Bilateral synchronous, slowly growing tumours are mostly PA and Warthin’s tumors. Diagnostic value of the parotid area palpation in multifocal bilateral tumors is limited, especially in those located in the deep lobe. Diffusion-weighted MRI is effective in differential diagnosis and identification of multifocal or/and bilateral parotid gland tumors.

The optimal treatment for BCA of the parotid gland is a superficial parotidectomy with facial nerve preservation. Total conservative parotidectomy is performed in cases of the tumors affecting the deep lobe. BCA may occur multifocally with the presence of even very small lesions. Parotidectomy provides lower recurrence rates compared to extracapsular tumor excision or enucleation [19].

Bilateral basal cell adenomas can be associated with coexisting dermal cylindromas. Most of BCA’s reported in literature occurred synchronously with dermal cylindromas [14, 16]. In our case, no dermal cylindromas were found.

Histologically, basal cell adenoma has four patterns: solid, trabecular, tubular and membranaceous. The membranaceous type (10% of BCA’s) is associated with a high recurrence rate of up to 25%. [20] The reason for this is the presence of pseudopodia and the lack of a complete capsule [21]. Malignant transformation is very rare (4%) and occurred mostly in membranaceous type [18].

Basal cell adenomas are uncommon tumours of salivary glands. Our study demonstrates that a careful diagnosis is essential to avoid overlooking multiple lesions in the same or contralateral gland once BCA is detected and to achieve possibly the most accurate pre-treatment diagnosis. Extracapsular excision or enucleation, that is frequently applied in Warthin tumors should not be performed in case of adenoma, which require conservative parotidectomy. Diffusion- and perfusion weighted magnetic resonance imaging is the most effective and safe visualization method of parotid gland tumours and should be considered when BCA is suspected.

## Data Availability

The datasets used and/or analysed during the current study are available from the corresponding author on reasonable request. All data generated or analysed during this study are included in this published article.

## Abbreviations

SGTs: Salivary gland tumors
WHO: World Health Organisation
BCA: Basal cell adenoma
MRI: Magnetic resonance imaging
DWI: Diffusion-weighted imaging
ADC: Apparent diffusion coefficient
PA: Pleomorphic adenoma
EMA: Epithelial membrane antigen
SMA: Smooth muscle actin
